# Changes in parafoveal retinal thickness and subfoveal choroidal thickness in a patient with dengue fever-associated maculopathy

**DOI:** 10.1186/1869-5760-3-63

**Published:** 2013-10-31

**Authors:** Kazuhiro Yamamoto, Hidenori Takahashi, Mikiko Kanno, Yasuo Noda, Yujiro Fujino

**Affiliations:** 1 Department of Ophthalmology, University of Tokyo, 7-3-1 Hongo Bunkyo-ku, Tokyo 113-8655, Japan; 2 Department of Ophthalmology, Jichi Medical University, 3311-1 Yakushiji Shimotsuke-shi, Tochigi 329-0431, Japan; 3 Department of Ophthalmology, Kawakita General Hospital, 1-7-3 Asagaya Suginami-ku, Tokyo 166-0001, Japan; 4 Department of Ophthalmology, Tokyo Kosei Nenkin Hospital, 5-1 Tsukudocho Shinjuku-ku, Tokyo 162-8543, Japan

**Keywords:** Dengue fever-associated maculopathy, Choroidal thickness, Retinal thickness, Optical coherence tomography

## Abstract

**Background:**

The time courses of retinal and choroidal thickness changes in dengue fever-associated maculopathy are not known. We measured central macular thickness (CMT), parafoveal retinal thickness (PRT), and subfoveal choroidal thickness (SCT), in one case, employing optical coherence tomography.

**Findings:**

The patient was a 43-year-old man diagnosed with dengue fever in Sri Lanka. He became aware of blurred vision bilaterally and visited our department 2 weeks after the onset. He showed reduced visual acuity. The Amsler test revealed a doughnut-shaped relative scotoma. Based on the course of this condition, dengue fever-associated maculopathy was diagnosed. One month later, 20 mg triamcinolone was injected into sub-Tenon space of the left eye. Both eyes showed subsequent improvement. PRT of both eyes increased 1 week after the first visit and decreased thereafter, reaching a plateau 1 month after the first visit. The change in CMT was minimal. SCT changed markedly, with the similar pattern in both eyes, though no particular tendency was noted during the observation period.

**Conclusions:**

Dengue fever-associated maculopathy appears to be closely associated with thickening of the inner layer of the retina, but not with changes in choroidal thickness.

## Findings

### Introduction

Dengue fever is a tropical endemic disease caused by dengue fever virus which is transmitted by the *Aedes aegypti* mosquito. In cases with severe infections, hemorrhagic dengue fever may develop. Dengue virus infection occurs in tropical and subtropical regions where the carrier mosquitoes are present. It has been estimated that worldwide, about 100 million individuals annually develop dengue fever, including about 250,000 with hemorrhagic dengue fever [1].

Ocular complications also develop in dengue fever [2]. The incidences of ocular complications were reported to be 119 (7.1%) of 1,686 patients with dengue fever and 18 (54.5%) of 33 patients with hemorrhagic dengue fever [3].

Ocular complications of dengue fever include retinal hemorrhages, maculopathy, uveitis, and optic neuritis [4]. Retinal hemorrhage or a Roth spot is often seen in the macular region and/or the surrounding area, and often develops when the platelet count is low, suggesting thrombocytopenia to be responsible for this complication [5]. Maculopathy with dengue fever is microangiopathy of the macular region, characterized by hemorrhages and soft exudates of the macula [6]. Furthermore, edema involving the inner layer of the retina was previously demonstrated employing optical coherence tomography (OCT) [7,8]. However, the time courses of retinal and choroidal thickness changes in maculopathy are not known. We recently encountered a patient who suffered from dengue fever-associated maculopathy and analyzed time-course data on retinal and choroidal thickness obtained employing OCT.

### Subjects and methods

**Figure 1:**
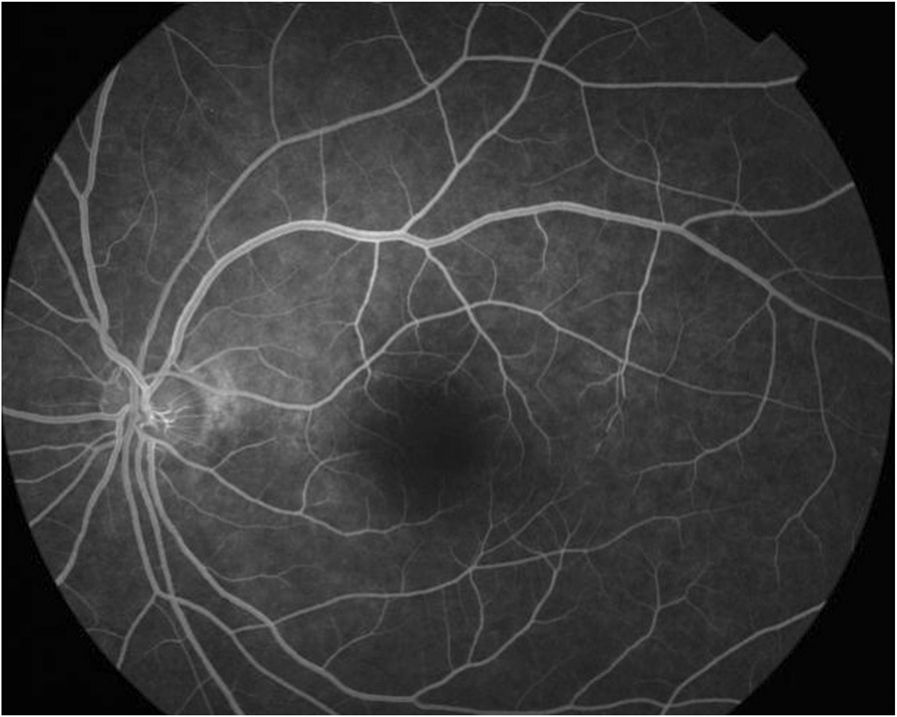
**Left fundus fluorescein angiography on day 14.** Slight leakage from the rim of the optic disc and slight fluorescence leakage in the macular area and interrupted macular venules are revealed.

**Figure 2:**
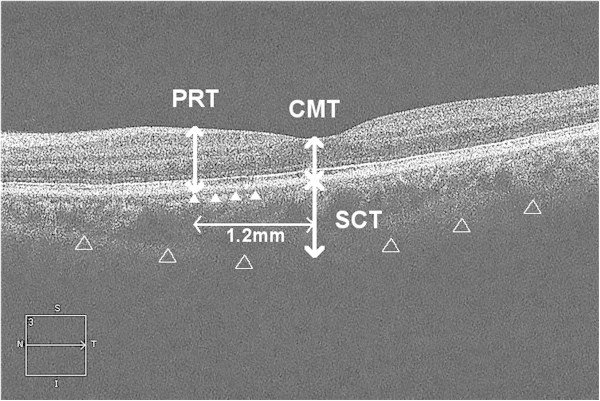
**Optical coherence tomography image of the left macula on day 34, obtained employing Cirrus HD-OCT Model 4000.** Indistinct photoreceptor IS/OS junction line (white arrowheads). The choroid-scleral border (white outlined arrowheads). CMT, central macular thickness; PRT, parafoveal retinal thickness; SCT, subfoveal choroidal thickness.

A 43-year-old Japanese man developed fever, headache, and diarrhea in August 2010, while in Sri Lanka. He was diagnosed as having dengue fever based on the dengue antibody test at Nawaloka Hospital (Colombo, Sri Lanka). In response to drip infusion therapy administered for 1 week, his systemic symptoms showed remission. On day 8 after disease onset, the patient became aware of blurred vision in both eyes. On day 11, he visited a local eye clinic, where he was found to have retinal hemorrhages. Since his eye condition did not improve, he returned to Japan and visited our hospital on day 14. At that time, his best corrected visual acuity had decreased to 0.8 in the right eye and 0.1 in the left eye accompanied by a reduction in critical flicker frequency (CFF) bilaterally (29 Hz in the right and 24 Hz in the left eye). The Amsler test, conducted at the first visit, revealed a doughnut-shaped relative scotoma, partially including an absolute scotoma. Fundus examination disclosed dot hemorrhages in the right eye. Fundus fluorescein angiography revealed slight leakage from the rim of the optic disc in both eyes and slight fluorescence leakage in the macular area, and blockage due to dot hemorrhages in the right eye as well as interrupted macular venules in the left eye (Figure 1). OCT demonstrated an indistinct photoreceptor inner segment/outer segment (IS/OS) junction line in both eyes (Figure 2). Static visual field testing (The Humphrey Field Analyzer central 30-2 threshold test; Carl Zeiss Meditec AG, Oberkochen, Germany) disclosed no abnormalities. The patient consented for this case to be presented.

Since this patient had developed retinal hemorrhages and mild optic nerve disorders, as well as microangiopathy noted on undus fluorescein angiography shortly after the development of dengue fever, dengue fever-associated maculopathy was diagnosed. Ocular inflammation was mild, and thus, the patient was followed without active treatment. After 1 month of follow up without intervention, the right eye showed restoration of normal visual acuity (1.2) and CFF (38 Hz), and there were no subjective symptoms with improvement of the IS/OS line on OCT. However, the left eye showed no improvement in CFF (29 Hz) or in subjective symptoms other than impaired visual acuity (1.2). Therefore, 20 mg triamcinolone acetonide was injected into the sub-Tenon space of the left eye. Subsequently, the fundus findings of the left eye, CFF and subjective symptoms improved, with normalization approximately 2.5 months later. Furthermore, the IS/OS line, as visualized by OCT, was also restored. The patient has been followed periodically and remains free of signs of ocular dengue relapse.

We measured the central macular thickness (CMT), parafoveal retinal thickness (PRT), and subfoveal choroidal thickness (SCT) during the course of the disease employing Cirrus HD-OCT Model 4000 (Carl Zeiss Meditec AG). We used the HD 5 Line Raster protocol (Carl Zeiss Meditec AG). CMT was defined as the retinal thickness at the center of foveola. PRT was defined as the retinal thickness at 1.2 mm nasal from the center of foveola in an OCT raster scan through the foveola. A third person randomized the order of OCT scan images. One of the co-authors manually measured the retinal thickness from the inner limiting membrane to the Bruch's membrane, and choroidal thickness from the Bruch's membrane to the choroid-scleral border (Figure 2).

## Results

**Figure 3:**
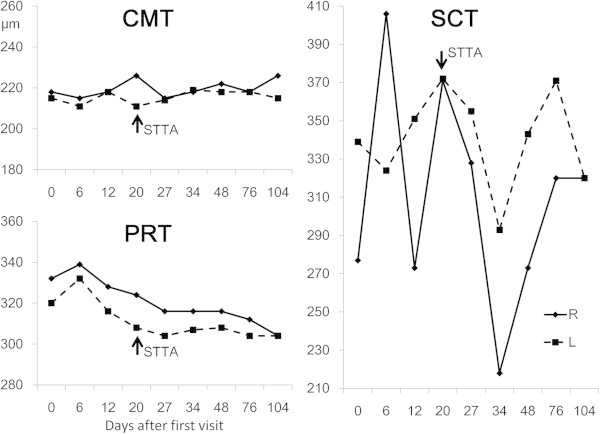
**Changes in central macular thickness, parafoveal retinal thickness, and subfoveal choroidal thickness in this patient.** The solid line shows the right eye, and the dashed line shows the left eye. In both eyes, the PRT was increased 1 week after the first visit and decreased thereafter, reaching a plateau 1 month after the first visit. The change in SCT was larger than that in PRT and showed essentially the similar pattern in both eyes, though no particular tendency was noted during the observation period. CMT changed minimally, showing no particular tendency.

In both eyes, PRT was increased 1 week after the first visit (right 339 μm, left 332 μm) and decreased thereafter, reaching a plateau 1 month after the first visit (right 316 μm, left 304 μm). The change in SCT was larger than that in PRT and showed essentially the similar pattern in both eyes, though no particular tendency was noted during the observation period. The mean thickness of SCT was 310 μm in the right eye and 341 μm in the left eye. CMT changed minimally, showing no particular tendency, on average 220 μm in the right eye and 215 μm in the left eye (Figure 3).

## Discussion

Our present patient had the characteristic ocular dengue findings of retinal hemorrhages, maculopathy and optic neuritis, accompanied by a scattered distribution of dotted retinal hemorrhages in the area showing a doughnut-shaped relative scotoma on the Amsler test. An unclear IS/OS line was also detected in the same area by OCT. Teoh et al. categorized maculopathy associated with dengue fever into 3 groups based on OCT imaging patterns [7]. Type 1 was diffuse retinal thickening with increased retinal thickness around the central/paracentral fovea. Type 2 was cystoid macular edema characterized by large intraretinal cystoid-like spaces. Type 3 was foveolitis with or without macular edema, characterized by an area of thickening and high reflectivity at the level of the subfoveal outer retina. Our case appears to be consistent with Type 3. In previous reports as well, there were cases with an unclear outer retinal layer due to block of OCT laser by edema of the inner layer of the retina. This suggests that the unclear IS/OS line seen in the present case reflects mild edema of the inner layer of the retina.

In this patient, the minimal change in CMT may reflect the outer retinal layer not being affected by dengue fever-associated maculopathy. PRT in both eyes peaked at 3 weeks after the development of dengue fever and decreased during the subsequent 4 weeks, remaining unchanged thereafter. The change in PRT is thought to reflect edema of the inner retinal layer. The SCT changes in the right and left eyes showed essentially a similar pattern, but there was no particular tendency during the observation period. Because of the similar pattern, major changes in SCT possibly reflect the systemic condition changes in dengue fever, rather than maculopathy.

Circadian changes in SCT have been recognized [9]. In this study, all the OCT images were obtained in the morning. It is further recommended that reports from endemic areas, reviewing many cases with this rather rare condition, should be analyzed.

## Abbreviations

CFF: Critical flicker frequency; CMT: Central macular thickness; IS/OS: Inner segment/outer segment; OCT: Optical coherence tomography; PRT: Parafoveal retinal thickness; SCT: Subfoveal choroidal thickness.

## Competing interests

The authors declare that they have no competing interests.

## Authors' contributions

KY conceived the study and its design, acquired the data, drafted the article, critically revised the paper for important intellectual content, and obtained funding. HT conceived the study and its design; acquired, analyzed, and interpreted the data; critically revised the manuscript for important intellectual content; contributed to statistical analysis; obtained funding; provided administrative, technical, or material support; and supervised the whole course of the study. MK and YN conceived the study and its design, acquired the data, obtained funding, and provided administrative, technical, or material support. YF conceived the study and its design, analyzed and interpreted the data, critically revised the manuscript for important intellectual content, obtained funding, provided administrative, technical, or material support, and supervised the study. All authors read and approved the final manuscript.

## Authors' information

All authors are ophthalmologists and had worked at Tokyo Kosei Nenkin Hospital from April 2009 to March 2011 together.
